# In-situ forming injectable GFOGER-conjugated BMSCs-laden hydrogels for osteochondral regeneration

**DOI:** 10.1038/s41536-022-00274-z

**Published:** 2023-01-06

**Authors:** Mi Yeon Ha, Dae Hyeok Yang, Su Jung You, Hyun Joo Kim, Heung Jae Chun

**Affiliations:** 1 Department of Biomedicine & Health Sciences, College of Medicine, The Catholic University of Korea, Seoul, 06591 Republic of Korea; 2 Institute of Cell and Tissue Engineering, College of Medicine, The Catholic University of Korea, Seoul, 06591 Republic of Korea; 3 Department of Medical Life Sciences, College of Medicine, The Catholic University of Korea, Seoul, 06591 Republic of Korea

**Keywords:** Tissue engineering, Regenerative medicine

## Abstract

The collagen-mimetic peptide GFOGER possesses the chondrogenic potential and has been used as a cell adhesion peptide or chondrogenic inducer. Here, we prepared an injectable in situ forming composite hydrogel system comprising methoxy polyethylene glycol-*b*-polycaprolactone (MPEG-PCL) and GFOGER-conjugated PEG-PCL (GFOGER-PEG-PCL) with various GFOGER concentrations based on our recently patented technology. The conjugation of GFOGER to PEG-PCL was confirmed by ^1^H NMR, and the particle size distribution and rheological properties for the sol-gel transition behavior of the samples with respect to the GFOGER content were evaluated systemically. In vitro experiments using rat bone marrow-derived mesenchymal stem cells (BMSCs) revealed that the GFOGER-PEG-PCL hydrogel significantly enhanced expression of integrins (β1, α2, and α11), increased expression of FAK, and induced downstream signaling of ERK and p38. Overexpression of chondrogenic markers suggested that BMSCs have the potential to differentiate into chondrogenic lineages within GFOGER-PEG-PCL samples. In vivo studies using a rat osteochondral defect model revealed that transplanted BMSCs with GFOGER_0.8_-PEG-PCL survived at the defect with strong chondrogenic expression after 4 weeks. The stem cell-laden GFOGER_0.8_-PEG-PCL hydrogel produced remarkable osteochondral regeneration at 8 weeks of transplantation, as determined by histological findings and micro-CT analysis. The histomorphological score in the GFOGER_0.8_-PEG-PCL + BMSCs group was ~1.7-, 2.6-, and 5.3-fold higher than that in the GFOGER_0.8_-PEG-PCL, MPEG-PCL, and defect groups, respectively. Taken together, these results provide an important platform for further advanced GFOGER-based stem cell research for osteochondral repair.

## Introduction

Articular cartilage has limited regeneration potential not only because this connective tissue does not contain blood vessels, but also because chondrocytes, the main cell types in cartilage, are bound in lacunae; once injured, cartilage heals with difficulty^1–3^. Therefore, over recent decades, tissue engineering that incorporates biology, medicine, and engineering has led to the development of therapeutic approaches to postpone the need for artificial joint replacement^4–6^. The success of tissue engineering depends on a harmonious interplay of three components: cells for neo-tissue formation, biomaterials to act as scaffolds, and biological signaling molecules that instruct cells to form the desired tissue type^7–10^. Among the components, scaffolds play a pivotal role in the field of modern regenerative medicine because they provide an architectural context in which cells and growth factors can cooperate and exhibit a wide range of morphological and geometric possibilities for clinical applications^8,11^. Many biomaterials have been adapted for the manufacture of scaffolds and are under study^7,8,12,13^.

Hydrogels have been widely applied in the field of cartilage tissue engineering because of their structural similarity to native cartilage extracellular matrix (ECM)^5,9,14^. Injectable in situ forming hydrogels, in particular, have gained great attention for their potential in cartilage regeneration^14,15^. The precursor solutions injected in vivo undergo gelation which may prevent the leakage of solution. Moreover, the solutions can conform to and support irregularly shaped wound sites^16,17^. These advantages have led to increasing use of in situ forming hydrogels in the field of minimally invasive surgery^18^.

The diblock copolymer methoxy polyethylene glycol-*b*-polycaprolactone (MPEG-PCL) is an ideal candidate material for in situ hydrogel scaffold in cartilage tissue engineering^14^. The phase transition of MPEG-PCL occurs near body temperature without any cross-linkers^19,20^. In addition, the in situ forming MPEG-PCL hydrogel has shown excellent retention capabilities for mesenchymal stem cells (MSCs)^21–23^. A drawback of MPEG-PCL is the lack of a cell recognition site for focal adhesion to orchestrate various aspects of cellular behaviors for development and maintenance of tissues^22^. The tripeptide RGD is a representative motif that is highly effective at promoting attachment of various cell types^7,18,22^. Other cell-adhesive peptide sequences, such as YIGSR, IKVAV, and GFOGER, have been identified from native ECM components. These ligands bind distinct receptors from the integrins and activate intracellular signaling pathways^8,24–26^. The collagen-mimetic ligand Gly-Phe-Hyp-Gly-Glu-Arg (GFOGER) possesses osteogenic and chondrogenic potential and has been used as a cell adhesion peptide or chondrogenic inducer in numerous studies^27,28^. Interestingly, the GFOGER sequence has been incorporated into biodegradable hydrogels, confirming its ability to support chondrogenic differentiation of MSCs, with increased expression and deposition of type II collagen and GAGs^28^.

**Fig. 1:**
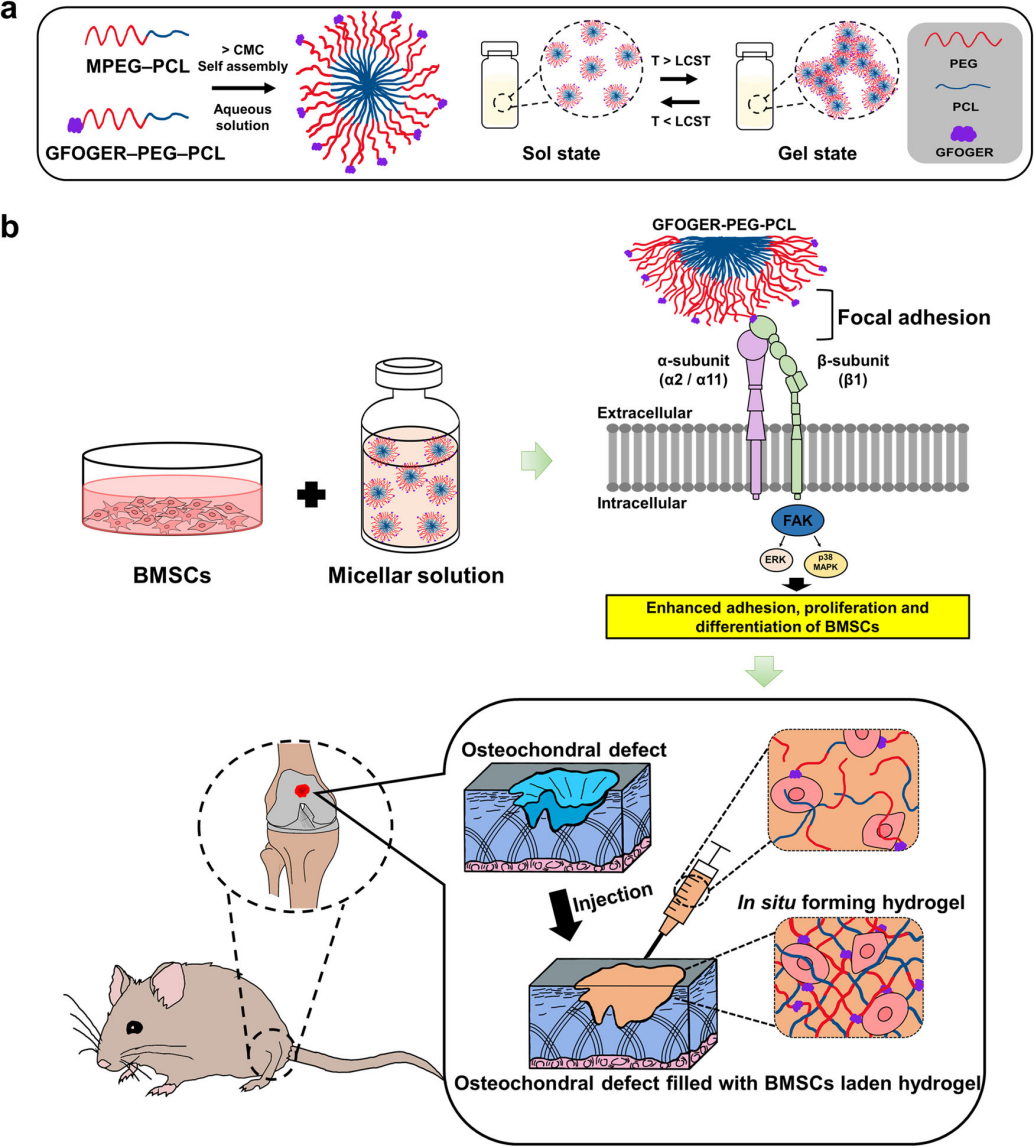
A schematic illustration of the experimental design. **a** PEG based diblock copolymers self-assemble into micelles in water at room temperature and a schematic showing the sol-gel phase transition mechanisms in aqueous solution. CMC critical micelle concentration, LCST lower critical solution temperature, T temperature. **b** Overview of the integrin signaling through BMSCs cultured in GFOGER-conjugated hydrogel and the applications of injectable hydrogel for rat osteochondral regeneration.

In this study, we propose an in situ forming hybrid hydrogel comprising MPEG-PCL and GFOGER-conjugated PEG-PCL (GFOGER-PEG-PCL) that undergoes gelation at a temperature above the lower critical solution temperature (Fig. 1a). The resulting hydrogel enhanced the focal adhesion between cellular integrins and the recognizable site to which the GFOGERs are conjugated. The immediate in situ gelation of precursor solution after administration into the cartilage defect allows the application of a minimally invasive surgical technique to repair osteochondral defects (Fig. 1b).

To this end, GFOGER was conjugated to the end group of PEG-PCL, and a series of precursor solutions of varying molar ratios of GFOGER (0.4–3.2 mM) was prepared (GFOGER_0.4-3.2_-PEG-PCL). The gel formation capability of these precursor solutions was assessed using dynamic light scattering (DLS) and viscosity measurements. In vitro experiments were conducted to evaluate the effects of GFOGER on cellular responses of BMSCs. The expression of integrins (β1, α2, and α11), focal adhesion kinase (FAK)/phosphorylated FAK (pFAK), extracellular signal-regulated kinase (ERK)/phosphorylated ERK (pERK), and p38 mitogen-activated protein kinase (p38)/phosphorylated p38 (pp38) was investigated. Additionally, hybrid hydrogels were evaluated for therapeutic efficacy in vivo with a critical-sized osteochondral defect rat model. A GFP-based tracing technique was used to examine the behavior of BMSCs transplanted within the defect site. Regenerated sites were assessed through histological staining and micro-computed tomographic (micro-CT) analyses. This is the study to investigate the ability of in situ forming injectable GFOGER-PEG-PCL hydrogels in osteochondral regeneration based on our recent patent^29^.

## Results and discussion

### ^1^H NMR characterization of GFOGER-PEG-PCL

**Fig. 2:**
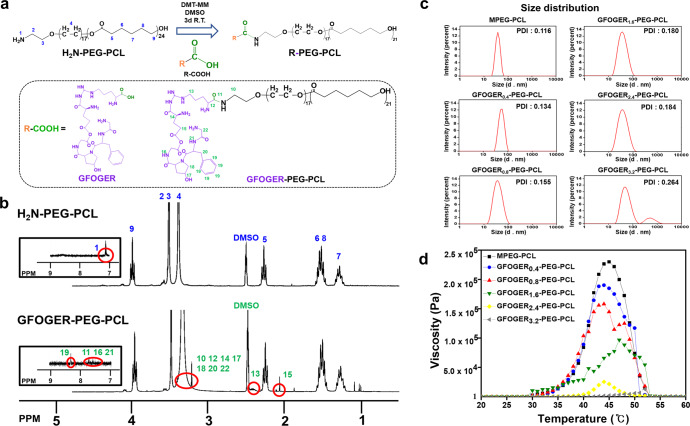
Synthesis and characterization of GFOGER-PEG-PCL. **a** Synthetic scheme of GFOGER-PEG-PCL. Conjugation of GFOGER via condensation reaction using DMT-MM as a condensing agent. **b** ^1^H NMR spectra of GFOGER-PEG-PCL polymer measured in DMSO-_d6_. **c** Particle size distribution of copolymer micelles in water measured by DLS at 25 °C. **d** Viscosities of MPEG-PCL, GFOGER_0.4_-PEG-PCL, GFOGER_0.8_-PEG-PCL, GFOGER_1.6_-PEG-PCL, GFOGER_2.4_-PEG-PCL, and GFOGER_3.2_-PEG-PCL hydrogel precursor solutions monitored from 20 to 60 °C by rheometer.

Conjugation of GFOGER to H_2_N-PEG-PCL was confirmed by ^1^H NMR analysis using DMSO-_d6_ (Fig. 2b). The signals at 3.40 and 3.50 ppm were assigned to the methyl group of PEG. The peaks at 3.98–4.02, 2.27–2.32, 1.50–1.62, and 1.26–1.36 ppm were attributed to PCL. The peaks related to GFOGER were observed at 8.45, 7.54–7.75, 3.23–3.43, 2.40–2.46, and 2.08 ppm. The signals appeared at 3.98–4.02 and 7.54–7.75 ppm and were assigned to the methyl group of PCL and phenylalanine of GFOGER, respectively. The integration ratio of H_2_N-PEG-PCL and GFOGER was 95%, which indicated that GFOGER was conjugated to H_2_N-PEG-PCL at a 1:1 M ratio.

### Size distribution and rheological properties of the diblock copolymer suspensions

To measure the mean diameter of micelles, the DLS technique was employed (Fig. 2c). MPEG-PCL showed a narrow size distribution with a PDI of 0.116. As the content of GFOGER increased from 0.4 to 2.4 mM (GFOGER_0.4-2.4_-PEG-PCL), the PDIs of micelles shifted to larger values and became broader. This implies the incorporation of two copolymers of MPEG-PCL and GFOGER-PEG-PCL into the same micelle at the molecular level^30^. However, introduction of large amounts of GFOGER (GFOGER_3.2_-PEG-PCL) resulted in difficulty in integration from the molecularly mixed state, displaying a bimodal distribution consisting of a population of small micelles and larger copolymer aggregates, as reported in previous studies^22,30^.

The viscosities of the hydrogel precursor solutions were monitored as a function of temperature ranging from 20 to 60 °C to investigate the sol-gel phase transition behaviors of the samples (Fig. 2d). At low temperatures, the solutions had a viscosity around 1 Pa, indicating that their sol states remained unchanged. As the temperature increased, the viscosity of the sample increased, and the final viscosities of MPEG-PCL, GFOGER_0.4_-PEG-PCL, GFOGER_0.8_-PEG-PCL, and GFOGER_1.6_-PEG-PCL were 2.3 × 10^5^, 1.8 × 10^5^, 1.5 × 10^5^, and 1.1 × 10^5^ Pa, respectively, all of which were in the range of 10^5^ Pa. Further increase in GFOGER content resulted in a sudden decrease in viscosity of the precursor solution. The final viscosity of GFOGER_2.4_-PEG-PCL was 2.0 × 10^4^ Pa, indicating failure of the sol-gel transition. Thus, GFOGER_2.4_-PEG-PCL was not a suitable sample for subsequent cell and tissue experiments.

The basic principle of MPEG-PCL micelle formation is that the hydrophobic PCL block constitutes a core due to the poor interaction between PCL and water, while the hydrophilic PEG block is hydrated to form an outer shell at the critical micelle concentration^31^. As temperature increases, both entropy and PEG-water interactions increase, causing PEG chains to unfold, which results in a gradual increase in micelle size^22^. At the sol-gel transition temperature, the hydrophilic PEG shell further extends and builds a bridge between other micelles, resulting in micellar packing^32^. However, the increase of a bulky GFOGER pendant group at the end of the PEG chain reduced the chance of interaction between PEGs. In addition, stearic hindrance by GFOGER interferes aggregation and packing between micelles for forming a denser gel^33^.

### Integrin expression in BMSCs-encapsulated hydrogel formulations

**Fig. 3:**
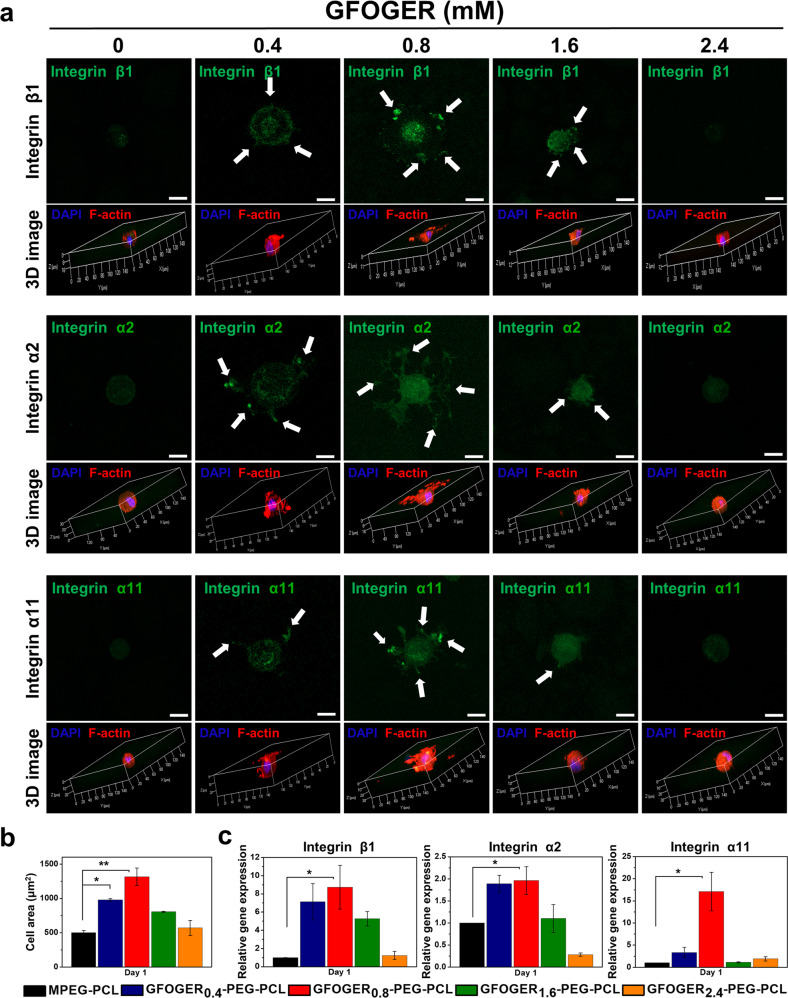
Evaluation of integrin expression and cell morphology induced by hydrogel formulations at day 1. **a** Immunofluorescence assay of integrins β1, α2, and α11 expressions (green, white arrows), F-actin (red) and nucleus (blue) (scale bar, 5 μm). And confocal laser scanning microscope 3D images of a cell morphology in hydrogels. **b** Quantification of cell spreading area analyzed based on 3D cell images of a cell morphology in hydrogels. **c** mRNA expressions of integrin β1, α2, and α11 of BMSCs cultured in hydrogels for 1 day (Data in (**b**, **c**) are shown as mean ± SD, unpaired Student’s *t*-test. *p < 0.05 and **p < 0.01).

On the basis of previous findings on GFOGER, the hydrogel samples prepared in this study have the capacity to positively affect MSC responses through interactions with integrins^8,24,25,34^. To examine the functional role of GFOGER-binding integrins related to focal adhesion in the hydrogel samples, BMSCs were immuno-stained and observed by confocal laser scanning microscopy. Figure 3a shows the expression of β1, α2, and α11 integrin at encapsulated cells. In the 0.8 mM GFOGER group, the strong expression of β1, α2, and α11 integrin was detected among samples. Moreover, three-dimensional (3D) images showed morphological changes in the cytoplasm of BMSCs. A radial filopodial growth pattern was evident in GFOGER_0.8_-PEG-PCL (Fig. 3a). Compared with the day 1 timepoint, each experimental group showed similar trend of cell morphology at day 3 and day 7. Especially, the 0.8 mM GFOGER group presented most spread cell morphology among the different concentration of GFOGER groups at day 1, day 3, and day 7 (Fig. 3a and Fig. S2a, S3a). Integrin β1, α2, and α11 staining exhibited similar pattern in accordance with the cell morphology data (Fig. 3a and Fig. S2a, S3a) and it has been confirmed with gene expression levels of integrins (Fig. 3c and Fig. S2c, S3c). In summary, the hydrogel-integrin interaction was greatest in the 0.8 mM GFOGER group, and it was persisted until day 7. Image analysis for quantifying cell area was performed based on the 3D cell images. The results showed that the cell areas were 502.44 ± 31.98, 979.33 ± 18.00, 1314.48 ± 128.149, 804.56 ± 7.99, and 569.74 ± 110.35 μm^2^. As a result, the widest cell area was shown in the 0.8 mM GFOGER group, as in the cell morphology image (Fig. 3b). To evaluate the time dependence of the cell morphology changes, the cell area of 3 and 7 days was also measured (Fig. S2b, S3b).

Quantification of β1, α2, and α11 integrin mRNA levels was carried out using qRT-PCR (Fig. 3c and S2c, S3c). The result did not show a linear increase according to GFOGER concentration. The highest expression of each integrin subunit was observed on the BMSCs-laden GFOGER_0.8_-PEG-PCL hydrogel among all samples. We presume that the larger is the number of ligands introduced to the surface of the substrate, the more likely they are to interact with the receptor. However, previous studies showed that closely packed ligands from excessive motif conjugation can create competition between multiple ligands for a single receptor and has the potential to limit access to receptors^35–37^. Therefore, we suggest that a GFOGER threshold concentration exists in cell response, and 0.8 mM of GFOGER could be an optimal concentration to maximize the integrin-mediated interaction between GFOGER-PEG-PCL hydrogel and BMSCs.

### Proliferation in BMSCs-laden hydrogel formulations

**Fig. 4:**
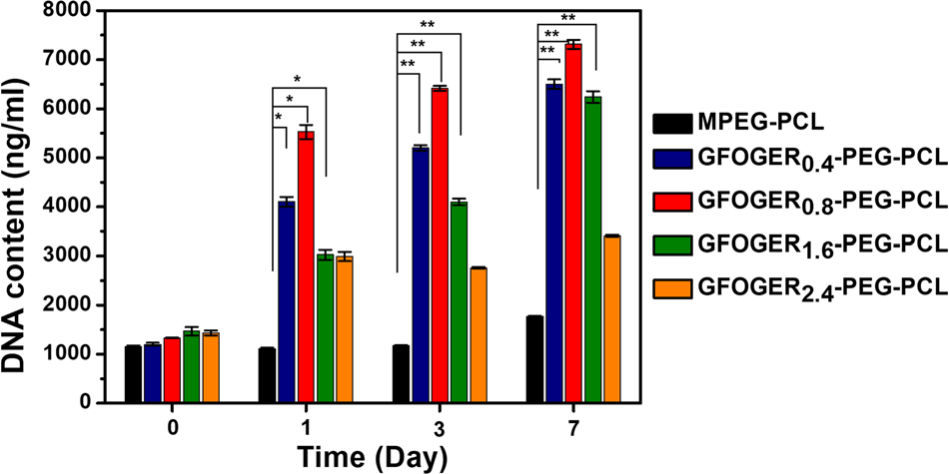
The influence of GFOGER on the proliferation of BMSCs. DNA quantification cultured BMSCs in hydrogels was assessed for 7 days with picogreen assay (Data were shown as mean ± SD, unpaired Student’s *t*-test. *p < 0.05 and **p < 0.01).

Cell proliferation within hydrogels was evaluated using the Picogreen assay, which assessed the DNA contents of the BMSCs-encapsulated hydrogel formulations for 7 days (Fig. 4). The initial DNA contents (day 0) of each sample were similar. During 7 days of culture, the amount of DNA gradually increased in the BMSCs-laden GFOGER_0.4-1.6_-PEG-PCL groups. Among them, the GFOGER_0.8_-PEG-PCL group showed the largest increase by approximately four-fold in DNA production compared with the control group.

### Integrin-mediated FAK-MAPK signaling pathway

**Fig. 5:**
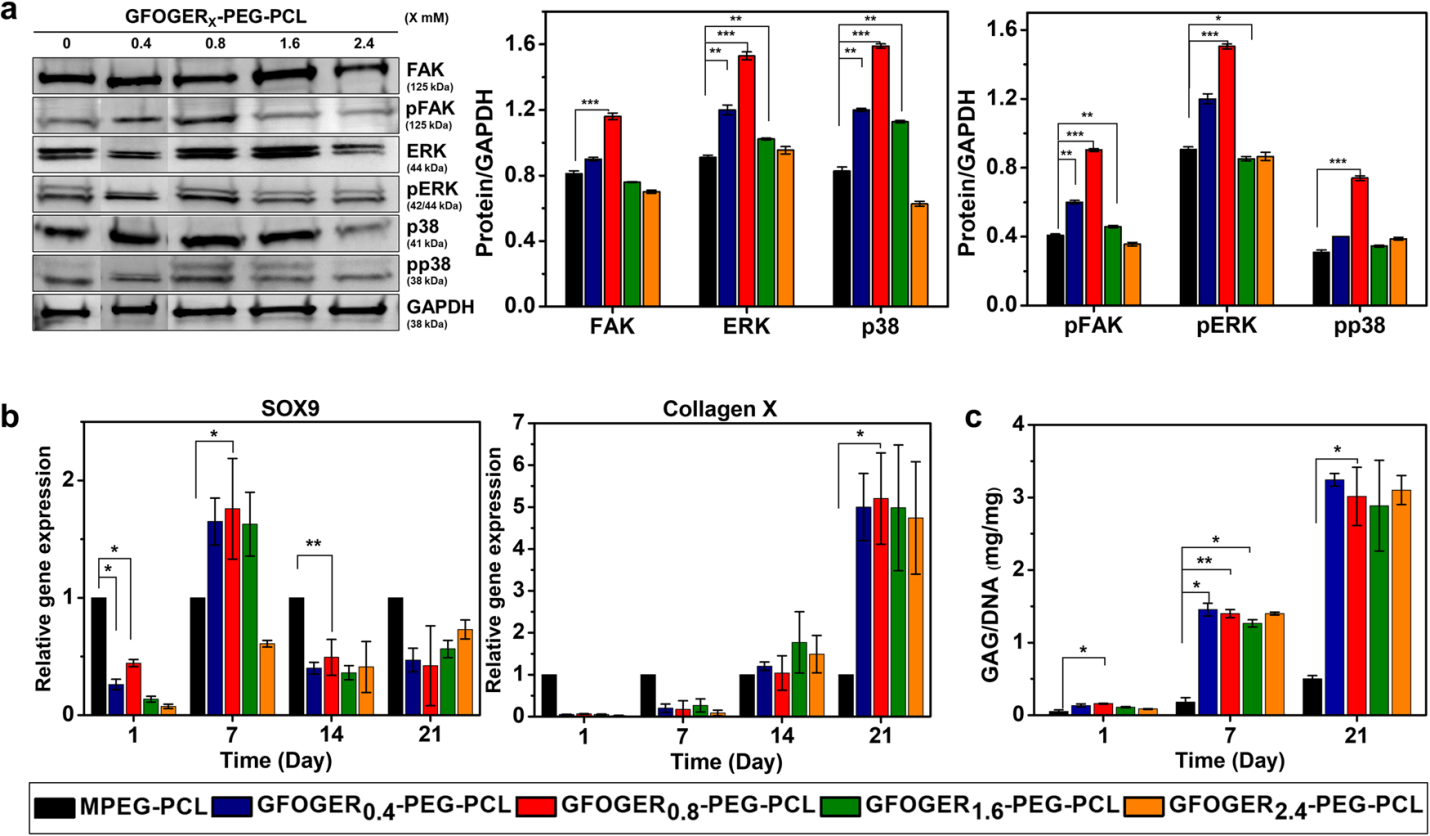
In vitro BMSC culture in the hydrogel composites. **a** Adhesion and proliferation of BMSCs in hydrogels activate FAK-MAPK signaling pathway. Cell lysates of BMSCs cultured for 24 h were prepared and western blot analysis with FAK, pFAK, ERK, pERK, p38, and pp38. **b** qRT-PCR for chondrogenic markers expression in hydrogels. BMSCs cultured in MPEG-PCL hydrogel were used as control and GAPDH was used as a reference gene. **c** GAG/DNA ratio where DNA was quantified using picogreen assay and GAG was quantified using the DMMB assay (Data are shown as mean ± SD, unpaired Student’s *t*-test. *p < 0.05, **p < 0.01, and ***p < 0.001).

The main role of integrin is to provide cells with focal adhesion and spreading ability with the ECM^38,39^. Stable ECM/integrin interactions induce FAK activation through phosphorylation, and the signals from the integrin-Src-FAK complex can be transmitted through or in crosstalk with the MAPK cascade (Ras-Raf-MEK-MAPK) involved in receptor tyrosine kinase (RTK)-mediated pathways^38,40^. Therefore, investigating the components of RTK-mediated signaling together with those of integrin-mediated signaling in BMSCs in hydrogels is of great significance to verify the correlation between the two pathways. Figure 5a shows the expression level of integrin via GFOGER in FAK-MAPK signaling pathway-related proteins analyzed of cultured cells for 24 h by western blot. The protein levels of FAK, ERK, and p38 increased in the presence of GFOGER. In particular, the levels of pFAK, pERK, and pp38 significantly increased in GFOGER_0.8_-PEG-PCL (0.90 ± 0.01, 1.50 ± 0.01, and 0.73 ± 0.01), indicating that signals from the GFOGER-integrin complex can be transmitted to downstream targets such as the FAK-MAPK (ERK/p38) cascade. This suggests that the capacity of GFOGER-conjugated PEG-PCL hydrogels to induce cellular proliferation is not only associated with induction of the integrin signaling pathway, but also controlled by growth- and proliferation-related pathways.

### Chondrogenic differentiation in BMSCs-laden hydrogel formulations

Many studies have indicated that MAPK signaling pathways lead to chondrogenic differentiation through activation of SRY-Box transcription factor 9 (SOX9)^41,42^. The subsequent activation of MAPKs (ERK/p38) in the integrin signaling cascade is critical for cell differentiation^38^. Therefore, we examined the expression of chondrogenic differentiation markers by RT-PCR and biochemical analysis to evaluate the chondrogenic potential of BMSCs cultured within hydrogel formulations (Fig. 5b, c). Chondrogenic differentiation was confirmed by evaluation of SOX9 gene expression as a marker of the early stage of chondrogenesis and collagen X gene expression as a downstream marker (Fig. 5b)^43^. In addition, biochemical analysis was carried out to determine the amount of GAG during chondrogenesis (Fig. 5c). At 7 days, there was an increase in the gene expression level of SOX9 in BMSCs-laden GFOGER_0.4-1.6_-PEG-PCL (1.65 ± 0.20, 1.76 ± 0.43, and 1.63 ± 0.27). At 21 days, collagen X gene expression was significantly upregulated in GFOGER_0.4-2.4_-PEG-PCL (5.00 ± 0.80, 5.20 ± 1.09, 4.98 ± 1.50, and 4.74 ± 1.34) compared with the control. GFOGER_0.8_-PEG-PCL hydrogel induced the highest gene expression of chondrogenic markers among all experimental groups. A marked increase of GAG secretion was observed in GFOGER-conjugated samples compared with controls throughout the chondrogenic differentiation period (Fig. 5c). Consequently, GFOGER_0.8_-PEG-PCL hydrogel, the sample with the threshold concentration for integrin expression, induced the highest expression of components of proliferation and differentiation signaling (MAPK/ERK) and the highest expression of SOX9, collagen X, and GAG.

### Histological observations

GFOGER_0.8_-PEG-PCL, which showed integrin-related cell signaling capacity in vitro and suitable in situ forming injectable hydrogel characteristics, was selected for further evaluation. We performed in vivo analyses by establishing an osteochondral defect rat model and examining five experimental groups: control, defect, MPEG-PCL, GFOGER_0.8_-PEG-PCL, and GFOGER_0.8_-PEG-PCL + BMSCs groups.

**Fig. 6:**
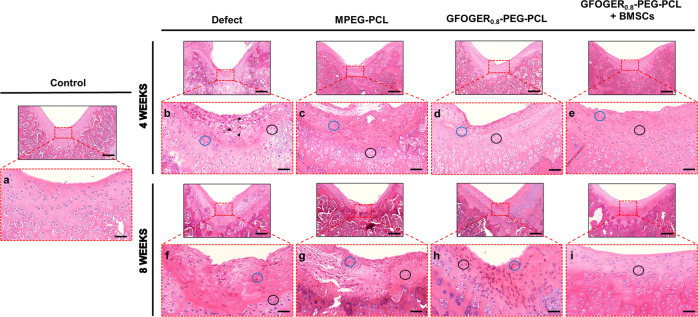
Representative H&E staining images of osteochondral defect tissue after 4 and 8 weeks of repair. The red frames indicate the magnified cartilage area (**a**–**i**). Chondrocyte in lacunae (black circles), chondroblast (blue circles), and inflammatory cells (arrowheads). Scale bar: 500 and 100 μm.

Figure 6 shows a cross-sectional images of H&E-stained rat cartilage from each group. Native articular cartilage (from control rats) is surrounded at the top by a layer of connective tissue known as the perichondrium that contains chondroblasts (round cells) with a few chondroprogenitor cells (elongated cells) (Fig. 6a). In the middle of the cartilage tissue, a large number of mature cartilage cells, chondrocytes, surrounded by lacunae was observed along with clusters of isogenous groups^37,44^. At 4 weeks of implantation, H&E staining revealed the infiltration of inflammatory cells and vascularization within the tissue in the defect group (Fig. 6b)^42^. In the MPEG-PCL - treated defect, few inflammatory factors were found compared with the defect group, and a number of precursor cells (elongated cells) was present together with fibrous matrices (Fig. 6c). The defect treated with GFOGER_0.8_-PEG-PCL showed a large number of chondroblasts on the top and chondrocytes at the bottom (Fig. 6d). In the BMSCs-transplanted defect, a large portion of the perichondrium was filled with chondroblasts, and a large numbers of chondrocyte filling the lacunae was found at the bottom (Fig. 6e). After 8 weeks post-implantation, as the inflammatory process dies down, a small number of progenitor cells was present in the tissue of the defect group but seemed like not to enough for regeneration or self-repair (Fig. 6f). The MPEG-PCL-treated sample showed an increased number of progenitor cells as well as chondroblasts at 8 weeks compared with 4 weeks (Fig. 6g). In the defect treated with GFOGER_0.8_-PEG-PCL, appositional growth was more profound compared with the defect and MPEG-PCL-treated defect (Fig. 6h). In particular, a large number of perichondrial cells was embedded in the central defect for expansion. The defect tissue transplanted with stem cells showed characteristics of the advanced stage of regeneration compared with tissue treated with other samples; chondral regeneration seemed to be near completion in the defect treated with GFOGER_0.8_-PEG-PCL + BMSCs (Fig. 6i). Progenitor cells were seen in the defects treated with other samples but were not observed in that treated with GFOGER_0.8_-PEG-PCL + BMSCs. Instead, the defect treated with GFOGER_0.8_-PEG-PCL + BMSCs contained a number of chondrocytes and, presumably, their precursor chondroblasts, along with many isogenous groups. The growth of cartilage in this case was dominated by the transplanted BMSCs in the GFOGER_0.8_-PEG-PCL hydrogel sample rather than through appositional growth starting at the outer edge of the cartilage; chondroblasts differentiated from BMSCs continued to secrete matrix components, promoting the formation of lacunae to develop into chondrocytes.

**Fig. 7:**
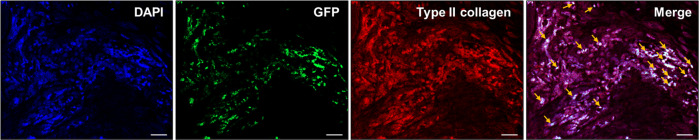
Immunofluorescence staining of GFP and type II collagen in BMSCs. GFP/type II collagen positive BMSCs were detected using immunofluorescence staining in the transplanted cartilage defect site at 4 weeks (yellow arrows). Scale bar: 20 μm.

In vivo tracing using immunohistochemical double staining provided important insights. As shown in Fig. 7, many GFP-transfected BMSCs were present at the defect site and were also stained with the chondrogenic marker for type II collagen. This offers indisputable evidence that transplanted BMSCs play a great role in chondrogenesis within defects.

**Fig. 8:**
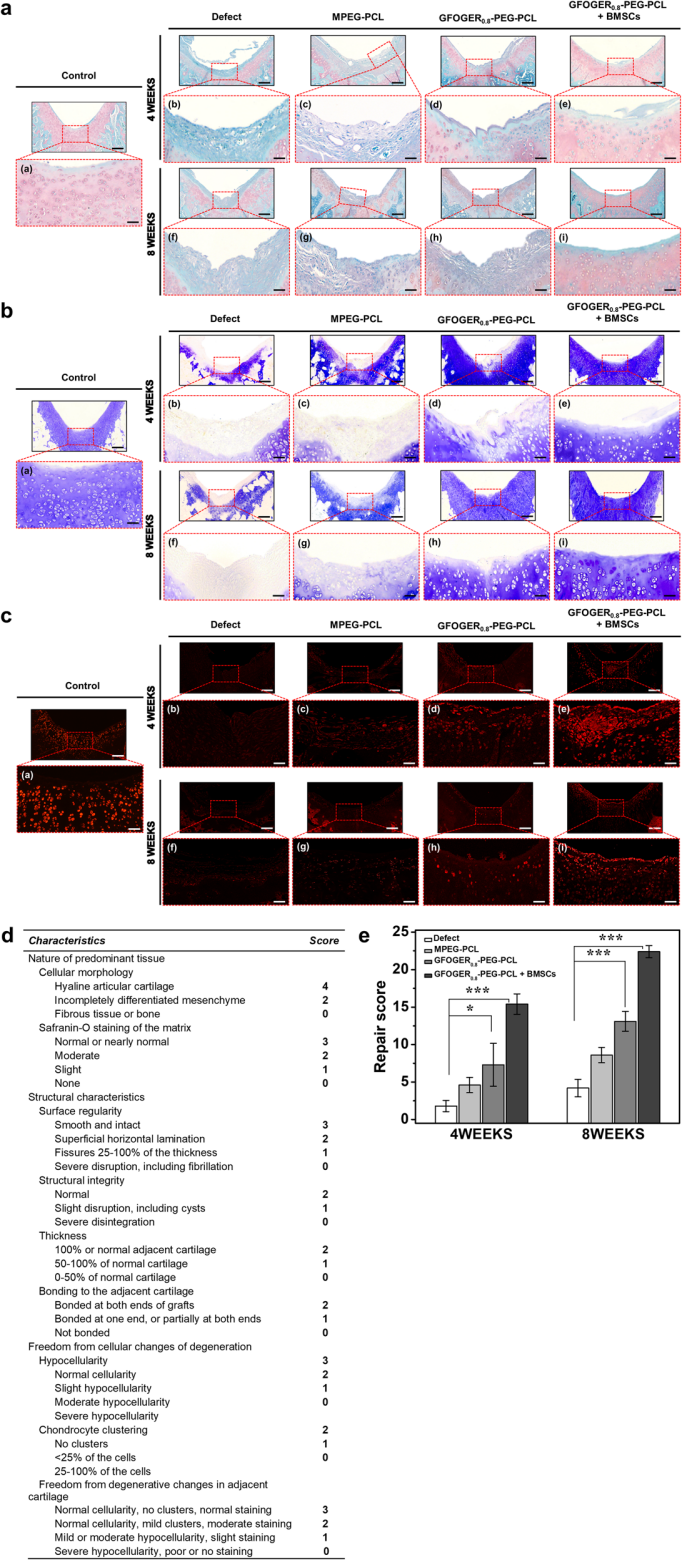
Histological analysis of osteochondral repair in rats. **a** Safranin O/fast green, **b** toluidine blue staining of cartilage defect tissue after 4 and 8 weeks of repair. Scale bar: 200 and 100 μm. **c** Immunofluorescence type II collagen staining of rat cartilage defect sites. Scale bar: 50 μm. And the red frames indicate the magnified cartilage area (a (a–i), b (a–i), and c (a–i)). **d**, **e** Macroscopic evaluation according to O’Driscoll histological scores (Data in (**e**) are shown as mean ± SD, unpaired Student’s *t*-test. *p < 0.05 and ***p < 0.001).

Figure 8 shows the formation of cellular matrix in the treated defects. Cartilage-specific staining including safranin O/fast green and toluidine blue were performed to evaluate GAG content in defect sites (Fig. 8a, b)^45^. The defects treated with BMSCs-laden hydrogel were stained intensely with safranin O and toluidine blue, which indicates the presence of an ECM rich in sulfated proteoglycan (Fig. 8a (e, i) and 8b (e, i))^46,47^. However, samples without BMSCs revealed matrix with little or no proteoglycan staining (Fig. 8a (b-d, f-h) and 8b (b-d, f-h)). At 8 weeks, the BMSCs-laden hydrogel group exhibited distinct staining (orange to red and blue) (Fig. 8a (i) and 8b (i)). Notably, toluidine blue staining produced an evenly stained territorial matrix with normal levels of pericellular staining usually found in couples or tetrads in the defect treated with the BMSCs-laden sample (Fig. 8b (i))^48,49^.

Type II and I collagen in regenerated cartilage tissues were evaluated by immunofluorescence staining (Fig. 8c and Fig. S4). Similar to GAG, the highest expression of type II collagen was found in the defect treated with GFOGER_0.8_-PEG-PCL + BMSCs (Fig. 8c (e) and 8c (i)). As a result of immunofluorescence staining with type I collagen, it was not found in the control group and the hydrogel groups. However, in the defect group, it was confirmed even after 8 weeks (Fig. S4). Therefore, the defect group showed poor cartilage regeneration due to the presence of fibrocartilage^50^.

The histomorphology observations were semi-quantified using the modified O’Driscoll grading scores (Fig. 8d)^51,52^. The total scores in the GFOGER_0.8_-PEG-PCL + BMSCs group were 15.40 ± 1.36 (4 weeks) and 22.40 ± 0.80 (8 weeks), which were significantly higher than those in the defect group (1.80 ± 0.75 and 4.20 ± 1.17, respectively), the MPEG-PCL group (4.60 ± 1.02 and 8.60 ± 1.02), and the GFOGER_0.8_-PEG-PCL group (7.30 ± 2.86 and 13.10 ± 1.33) (Fig. 8e).

### Regeneration of articular cartilage and subchondral bone evaluated by micro-CT

**Fig. 9:**
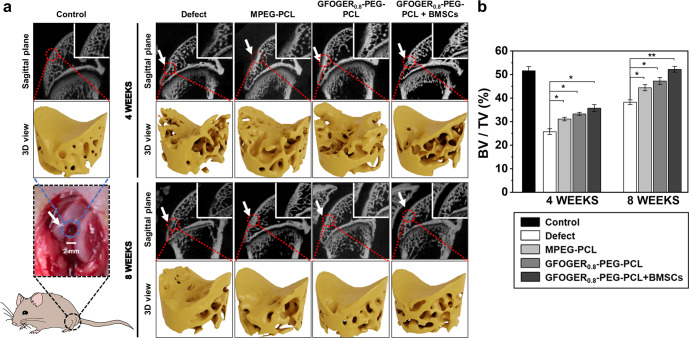
Micro-CT scanning. **a** Reconstructed micro-CT images in sagittal planes of the representative excised knee specimen from each experimental group at 4 and 8 weeks after implantation (White arrows indicate the defect site). And the white frames indicate the magnified defect area. 3D view of extracted bone from region of interest (ROI). **b** Quantitative analysis of reconstructed micro-CT images was used to analyze the ratio of bone volume to tissue volume (BV/TV) (Data in (**b**) are shown as mean ± SD, unpaired Student’s *t*-test. *p < 0.05 and **p < 0.01).

The two-dimensional (2D) sectioned (sagittal plane) and reconstructed 3D images of micro-CT strongly substantiated the histomorphological findings (Fig. 9). As shown in the 2D images (Fig. 9a), immature articular cartilage regeneration was still observed at all groups for 4 weeks of implantation. On the other hand, at 8 weeks, a different aspect of osteochondral regeneration was found at each group. Only peripheral repair around the host tissue was observed in the defect group. In the MPEG-PCL-treated defect, neo-tissue formation was found from the peripheral to focal point, but tissue formation was not dense enough. In contrast, the concave area of the GFOGER_0.8_-PEG-PCL-treated defect was filled with dense and integrated neo-cartilage and subchondral bone. Moreover, the results of micro-CT of the BMSCs transplanted sample were similar to those of the control.

Investigating subchondral bone formation in the osteochondral defect of animal model is an important factor in assessing articular cartilage regeneration because the defect involves both the cartilage and underlying bone^5,53^. The subchondral bone plate provides mechanical support for maintaining the structural integrity and nutrition supply to the overlying cartilage layer^13,54^. Therefore, if immature subchondral bone is produced, it affects the biomechanical properties of osteochondral region and shorten the lifespan of recovered osteochondral tissue^53^. For the 3D images at 4 weeks (Fig. 9a), the GFOGER_0.8_-PEG-PCL + BMSCs sample seemed to induce activated subchondral bone regeneration as compared to the defect, MPEG-PCL, and GFOGER_0.8_-PEG-PCL. In addition, MPEG-PCL and GFOGER_0.8_-PEG-PCL-treated defects were filled with more subchondral bone than the defect. To further investigate subchondral bone regeneration, BV/TV was calculated (Fig. 9b)^5^. A gradual increase in the BV/TV was observed at all groups for 8 weeks. Moreover, the GFOGER_0.8_-PEG-PCL + BMSCs showed the highest BV/TV among all groups throughout the period. At 4 weeks, the BV/TV percentages of defect, MPEG-PCL, GFOGER_0.8_-PEG-PCL, and GFOGER_0.8_-PEG-PCL + BMSCs groups were 25.75 ± 1.23, 31.11 ± 0.75, 33.26 ± 0.66, and 35.72 ± 1.47, respectively. At 8 weeks, the BV/TV percentages were 38.24 ± 0.98, 44.42 ± 1.30, 47.22 ± 1.50, and 52.23 ± 1.24, respectively.

Consequently, a series of in vivo studies demonstrated that the BMSCs-laden hydrogel sample had the best osteochondral regeneration capacity, which is the result of incorporation of GFOGER into the hydrogel, enabling long-term retention of BMSCs. Therefore, the present study provides noteworthy findings as a platform for further advanced GFOGER-based stem cell research for osteochondral regeneration.

## Methods

### Materials

We purchased ε-caprolactone (ε-CL), methoxy polyethylene glycol (MPEG; Mw: 750 g/mol), hydrogen chloride (HCl) solution (1.0 M) in diethyl ether, ethylene diamine (ED), and dimethyl sulfoxide-d_6_ (DMSO-_d6_) from Sigma Aldrich (MO, USA). H_2_N-polyethylene glycol-*b*-polycaprolactone (PEG-PCL) and GFOGER were purchased from creative PEG Works (NC, USA) and GL Biochem Ltd. (Shanghai, China), respectively. Dialysis tubes (Spectrum Laboratories Inc., NJ, USA) were used for purification of GFOGER-conjugated samples. We obtained 4-(4,6 dimethoxy-1,3,5-triazin-2-yl)-4-methylmorpholinium chloride (DMT-MM) from Wako Pure Chemical Industries (Osaka, Japan). Rat bone marrow-derived MSCs (BMSCs) of passage 2 were purchased from LONZA (Basel, Switzerland). All chemicals were used as received without further purification.

### Conjugation of GFOGER to H_2_N-PEG-PCL

Figure 2a shows a schematic illustration of the preparation of GFOGER-PEG-PCL. In previous studies, a cell-adhesive peptide was coupled to the end group of PCL via an *N*,*N*-carbonyldiimidazole-mediated amidation reaction^22,29^. In the present work, GFOGER was conjugated to the amine group at the end of PEG through a condensation reaction to couple to the outer shell as follows^33^. GFOGER (0.006 mmol, 4 mg) was conjugated to H_2_N-PEG-PCL (0.031 mmol, 100 mg) using DMT-MM (0.006 mmol, 1.66 mg) and was reacted in DMSO-_d6_ at room temperature (RT) for 3 days. The solution was purified using a dialysis tube (cutoff: 1 KD) for 3 days. The obtained solution was lyophilized at –90 °C for 7 days and stored in a freezer (–70 °C) for later use (GFOGER-PEG-PCL). Conjugation of GFOGER to H_2_N-PEG-PCL was confirmed by ^1^H NMR spectroscopy (Bruker ULTRASHIELD 300; Bruker, MA, USA) using DMSO-_d6_.

### Preparation of MPEG-PCL- and GFOGER-modified PEG-PCL hydrogel precursor solutions

A series of hydrogels consisting of various contents of MPEG-PCL and GFOGER-PEG-PCL was prepared to examine the effect of pendant GFOGER on gel formation. Hydrogel precursor solutions were prepared by mixing 20 w/v% of MPEG-PCL- and GFOGER-conjugated PEG-PCL (0.125, 0.25, 0.5, 0.75 and 1 w/v%, corresponding to 0.4, 0.8, 1.6, 2.4, and 3.2 mM of GFOGER, respectively) into a total volume of 1 ml in Dulbecco’s phosphate-buffered saline (DPBS). The respective formulations were designated as MPEG-PCL, GFOGER_0.4_-PEG-PCL, GFOGER_0.8_-PEG-PCL, GFOGER_1.6_-PEG-PCL, GFOGER_2.4_-PEG-PCL, and GFOGER_3.2_-PEG-PCL, where the numbers depict the concentrations of GFOGER. After phase separation in a water bath at 80 °C, the precursor solutions were vortexed to form a homogenous dispersion and stored at 4 °C for 2 days prior to hydrogel preparation. Table 1 lists the detailed compositions of MPEG-PCL and GFOGER_0.4-3.2_-PEG-PCL.

**Table 1:** Details of the quantity MPEG-PCL and GFOGER-PEG-PCL used for preparing the hydrogel blend solutions.

Samples	MPEG-PCL (w/v%)	GFOGER-conjugated PEG-PCL (w/v%)	GFOGER (mM)	DPBS (mL)
**MPEG-PCL**		0	0	
**GFOGER**_**0.4**_**-PEG-PCL**		0.125	0.4	
**GFOGER**_**0.8**_**-PEG-PCL**		0.25	0.8	
**GFOGER**_**1.6**_**-PEG-PCL**	20	0.5	1.6	1
**GFOGER**_**2.4**_**-PEG-PCL**		0.75	2.4	
**GFOGER**_**3.2**_**-PEG-PCL**		1	3.2	

### Dynamic light scattering (DLS) and rheology for viscosity measurement

The mean diameter and polydispersity index (PDI) of micelles formed in hydrogel precursor solutions were characterized by DLS (Zetasizer Nano ZS; Malvern Instruments, Malvern, UK)^55^. A specific concentration of solution (2 mg/mL) was immersed in Polystyrene Disposable Cells (Q-VETTES Semi-micro; Ratiolab, Frankfurt, Germany) and measured at 25 °C. The DLS analysis program ran through five cycles. The viscosity was measured using a rheometer (AR 2000 ex; TA Instruments, DE, USA) at temperatures from 20 to 60 °C.

### Cell culture

Rat BMSCs (LONZA; Basel, Switzerland) were expanded in MSCBM culture medium (LONZA; Basel, Switzerland) at 37 °C, 5% CO_2_ for in vitro experiments. GFP-transfected BMSCs (Cyagen Biosciences; Jiangsu, China) were cultured in MSC growth medium (Cyagen Biosciences) for in vivo experiments. The medium was changed every 3 days. Confluent cells were passaged using trypsin 0.25% EDTA in HBSS (Gibco, MA, USA) for up to six passages.

### Cell seeding in hydrogels

The cells were dispersed in 10 μl of medium and added to 50 μl of MPEG-PCL and GFOGER-PEG-PCL solutions. The cell density was 1 × 10^6^ cells/ml. The cell-containing precursor solutions were transferred to 96-well plates and kept in a humidified incubator at 37 °C, 5% CO_2_.

### Cell morphology and integrin expression

The attachment and spreading of BMSCs inside the hydrogel samples were monitored after 1, 3, and 7 days in culture by staining for integrins (β1, α2, and α11) and F-actin filaments with phalloidin. Samples were rinsed with DPBS and fixed in 4% formaldehyde (Samchun, Seoul, Republic of Korea) in DPBS for 10 min. Fixed cells were permeabilized with 0.1% Triton X-100 (Sigma Aldrich) in DPBS for 5 min. The cells were incubated with integrin β1 antibody (ab78502, dilution ratio 1:100, Abcam, Cambridge, UK), integrin α2 antibody (sc-53353, dilution ratio 1:100, Santa Cruz Biotechnology, TX, USA), and integrin α11 antibody (sc-390091, dilution ratio 1:100, Santa Cruz Biotechnology) overnight at 4 °C. The secondary antibody DyLight 488® goat anti-mouse IgG H&L (ab96879, Abcam, Cambridge, UK) was used at a 1:500 dilution in DPBS for 1 h. Phallotoxin (Invitrogen, MA, USA) was diluted to 1:100 in DPBS. Phallotoxin solution-treated cells in hydrogel were incubated for 1 h at RT. Samples were stained with DAPI (Thermo Fisher Scientific, MA, USA) diluted 1:500 in DPBS for 5 min at RT. After three washes in DPBS, samples were stored at 4 °C. Images were obtained using the LSM 510 Meta imaging system (Zeiss, Oberkochen, Germany). To measure the cell area, z-stack max intensity projections of single cells were produced using F-actin immunofluorescence staining. Image J software (NIH, MD, USA) was used to calculate the cell spreading area^35^. In order to calculate relative pixel intensity per cell area values, relative intensity data were divided by the corresponding cell area.

### Quantitative realtime-polymerase chain reaction (qRT-PCR)

Integrin-mediated adhesion- and chondrogenic differentiation-related genes were quantified using qRT-PCR^43^. BMSCs were cultured in hydrogels for 1, 3, and 7 days in culture medium at 37 °C, 5% CO_2_. After stabilizing the cells, the culture medium was changed to differentiation medium. Cells were cultured for 1, 7, 14, and 21 days in chondrogenic differentiation medium (Stem Pro; Gibco) and washed with DPBS. Total RNA was extracted using TRIzol Reagent (Invitrogen) and the ReliaPrep^TM^ RNA Cell Miniprep System (Promega, WI, USA) according to the manufacturer’s instructions. The use of Phase Lock Gel (5PRIME GmbH, Hamburg, Germany) can increase the RNA yields of cells separated from hydrogel. RNA pellets were dissolved in 15 μl of RNase- and Nuclease-Free water. Total RNA was measured using a Nano Drop spectrophotometer (ND-1000; Thermo Fisher Scientific) and was reverse transcribed according to the protocol of the manufacturer. Briefly, 0.1 μg RNA was mixed with Oligo (dT) primer (Thermo Fisher Scientific), 5X Reaction Buffer (Thermo Fisher Scientific), RNase Inhibitor (Thermo Fisher Scientific), dNTP Mix (Thermo Fisher Scientific), RevertAid H Minus Reverse Transcriptase (Thermo Fisher Scientific), and DEPC-treated distilled water in a total volume of 20 μl. Reactions were run in a thermal cycler using a cycle of 60 min at 42 °C, 5 min at 95 °C and then at 4 °C. qRT-PCR was performed using a Real-Time PCR machine (CFX96 Touch; Bio-Rad, CA, USA) in 0.1 ml qPCR 8-Strip tubes (MB-q100; Gunster Biotech, New Taipei City, Taiwan) in a reaction volume of 10 μl. One microliter of each RT reaction was amplified in a 20 μl PCR assay volume containing 300 nM of primers (Tables 2) and 2X SYBR Green Supermix (Bio-Rad). Gene expression was normalized to that of GADPH mRNA and calculated using the 2^−ΔΔCT^ method.

**Table 2:** Primer for genes.

Genes	Accession Number	Forward primer (5′-3′)	Reverse primer (5′-3′)
***Integrin β1***	NM_017022.2	GAACTTGTTGGTCAGCAGCG	TGGAAAACACCAGCAGTCGT
***Integrin α2***	XM_345156.8	AAGCCACGCCTGAATTTGTTAC	CCAAGAACTTGTCAATACCCCC
***Integrin α11***	NM_001108156.1	AATGGCCACCAGAAGACAGG	CTGCAGGCATTGGACAGAGT
***SOX9***	NM_080403.2	CCAAGGGCAAGGAAAGGAGA	CAGGTGAAGGTCTGAGCTGG
***Collagen X***	XM_032888300.1	CCTGGTGATGCATATGGAGGT	TACCCACTGTTGCTGCTCAC
***GAPDH***	NM_017008.4	TTCACCACCATGGAGAAGGC	CTCGTGGTTCACACCCATCA

### Picogreen assay

BMSCs proliferation was quantified using the Quant-iT™ Pico Green™ dsDNA Reagent and kits (Invitrogen). BMSCs on hydrogels were rinsed twice using DPBS and transferred to 1.5 ml tubes. Samples were frozen at –80 °C, suspended in 700 μl of lysis buffer (200 mM Tris-HCl, 20 mM EDTA/ddH2O/1% Triton X-100), and incubated for 24 h at 60 °C. To ensure complete cell lysis, samples were vortexed. To separate the hydrogels, the samples were filtered using a 0.22 μm filter (Merck Millipore, MA, USA). Dilutions of samples were prepared in TE at 1:100, and 100 μl of the sample was plated in black 96-well plates (SPL, Gyeonggi, Republic of Korea) with 100 μl of a working solution of Quant-iT PicoGreen reagent (Thermo Fisher Scientific). The samples were incubated for 2–5 min at RT. Fluorescence readings were obtained in a Fluorometer (Synergy MX; BIO-TEK, VT, USA) at 480 nm (excitation) and 520 nm (emission). A standard curve was prepared using calf thymus DNA in serial dilutions in 1% (v/v) Triton X-100. A blank was used to correct the background absorbance, and the assay was performed in triplicate.

### Chondrogenic differentiation of BMSCs in hydrogels

BMSCs were encapsulated into hydrogel formulations at a density of 1 × 10^6^ cells/ml in 24-well culture plates at 37 °C, 5% CO_2_. After 24 h of incubation, BMSCs-encapsulated hydrogels were exposed to Stem Pro chondrogenic differentiation media (Gibco) and maintained for up to 21 days. The medium was replaced every 3 days until the experiment was terminated.

### Biochemical assay for glycosaminoglycan (GAG) production

During the chondrogenesis of BMSCs, medium was changed every 3 days. GAGs were quantified using the Sulfated Glycosaminoglycans Quantification kit (AMS Biotechnology, Abingdon, UK)^56^. Hydrogels were collected at 1, 7, and 21 days for assessment of GAG content and were dissolved in papain lysis buffer (20 mM sodium phosphate buffer at pH 6.8 containing 1 mM EDTA, 2 mM dithiothreitol, and 300 μg/ml papain) for 1 h at 60 °C. Chondroitin sulfate was diluted in water from 0.5 to 20 μg/ml and used to create the standard curve. A volume of 0.1 ml of each sample or standard was pipetted into a 96-well plate. A volume of 0.1 ml of the DMMB dye was added to each well, and the optical density was measured at 520 nm with an ELISA Reader (Power wave XS; BIO-TEK). Quantification of GAGs was normalized to DNA content for each sample. DNA quantity was measured at 1, 7, and 21 days using a Quant-iT™ Pico Green™ dsDNA Reagent and kits (Invitrogen) following the manufacturer’s instructions.

### Western blotting

Total protein was extracted with PRO-PREP^TM^ protein extraction solution (iNtRON Biotechnology, Gyeonggi, Republic of Korea) from BMSCs cultured on hydrogel for 24 h. Protein concentration was calculated using a Bradford assay kit (Bio-Rad). Samples (20 μg) were separated by sodium dodecyl sulfate polyacrylamide gel electrophoresis (SDS-PAGE) and transferred to polyvinylidene fluoride (PVDF) membranes (Millipore, MA, USA). The membrane was rinsed with tris-buffered saline containing 0.1% Tween-20 (TBST), followed by blocking with 5% skim milk in TBST. The membrane was incubated with primary antibodies against FAK (sc-558, dilution ratio 1:200, Santa Cruz Biotechnology, TX, USA), pFAK (sc-16662, dilution ratio 1:200, Santa Cruz Biotechnology), ERK (ab9363, dilution ratio 1:250, Abcam, Cambridge, UK), pERK (919301, dilution ratio 1:1000, Bio Legend, CA, USA), p38 (ab31828, dilution ratio 1:1000, Abcam), pp38 (ab4822, dilution ratio 1:1000, Abcam), and glyceraldehyde 3-phosphate dehydrogenase (GAPDH; MA5-15738, dilution ratio 1:1000, Thermo Fisher Scientific) at 4 °C overnight. The membranes were washed and incubated with secondary antibody (Anti-mouse IgG-HRP; 7076, dilution ratio 1:1000, Cell signaling) (Goat anti-rabbit IgG-HRP; sc-2004, dilution ratio 1:5000, Santa Cruz Biotechnology) (Goat anti-mouse IgG-HRP; sc-2005, dilution ratio 1:5000, Santa Cruz Biotechnology) (Rabbit anti-goat lgG H&L-HRP; ab6741, dilution ratio 1:5000, Abcam) conjugated to horseradish peroxidase (HRP) for 1 h at RT. After another wash, the membranes were analyzed using the Luminescent Image Analysis System (LAS-4000; Fuji film, Tokyo, Japan) and Clarity Western ECL Substrate (Bio-Rad). The signals were captured and analyzed using the Image Studio Lite software (LI-COR Biosciences, NE, USA). Gels and blots in a specific panel derive from the same experiment and were processed in parallel. Unprocessed and uncropped scans of blots shown in Fig. 5a are shown in Fig. S5.

### Ethics statement

The animal experimental procedures were approved by the Institutional Animal Care and Use Committee (IACUC) in Yeouido St Mary’s Hospital of the Catholic University of Korea (YEO-2019011-FA). All animal procedures were performed in accordance with the Animal Protection Act, the Guide for the Care and Use of Laboratory Animals for rodent experiments provided by the IACUC in Yeouido St Mary’s Hospital of the Catholic University of Korea.

### Animals

Sprague-Dawley (SD) male rats (9–10 weeks old) were purchased from Doo Yeol Biotech (Seoul, Republic of Korea). Prior to the experiments, all animals were housed in specific pathogen-free animal rooms.

### Surgical procedure

Details of the surgical procedures to establish the osteochondral defect rat model are shown in Fig. S1. The experimental animals were divided randomly into five groups: the control group (*n* = 2), the defect group (*n* = 5), the MPEG-PCL group (*n* = 5), the GFOGER-conjugated group (GFOGER_0.8_-PEG-PCL; *n* = 5), and the GFOGER-conjugated PEG-PCL with BMSCs group (GFOGER_0.8_-PEG-PCL + BMSCs; *n* = 5). The animals were housed in pairs for the duration of the experiments.

To induce the defect, SD rats were anesthetized using Zoletil (Virbac, Carros, France)/Rompun (Bayer, Leverkusen, Germany) administered by intraperitoneal (IP) injection in an IACUC-approved animal laboratory facility. After the knee was shaved and sterilized using povidone-iodine swabs, a medial parapatellar incision was created, and the patella was deflected laterally to expose the trochlear surface (Fig. S1a–c). A hole (2 mm in diameter) was drilled into the center of the trochlea using a Micro Motor Handpiece (STRONG 202 N; SAESHIN, Daegu, Republic of Korea) to induce an osteochondral defect (Fig. S1d, e). After the creation of the defect, MPEG-PCL hydrogels, GFOGER_0.8_-PEG-PCL hydrogels, or BMSCs encapsulated in GFOGER_0.8_-PEG-PCL were implanted by injection using a 1 ml syringe (SUNGSHIM, Gyeonggi, Republic of Korea) to achieve full defect filling (Fig. S1f). The patella was relocated physically, and the joint capsule and subcutaneous tissue were closed with Vicryl 5-0 suture (ETHICON, NJ, USA) (Fig. S1g). The skin was closed with a tissue adhesive bond (3 M, MN, USA) (Fig. S1h). At 4 and 8 weeks, the knees were harvested, and the samples were fixed in 10% formalin for analysis.

### Histological analysis of cartilage repair

Formalin-fixed samples were decalcified in Calci-Clear Rapid solution (National Diagnostics, GA, USA) for 24 h at RT. Specimens were dehydrated through increasing concentrations of ethanol, followed by paraffin embedding. Samples were sectioned at a thickness of 7-μm and stained with hematoxylin-eosin (H&E), safranin O, and toluidine blue according to standard protocols. Immunohistochemistry for GFP, type II collagen, and type I collagen in BMSCs-laden samples was performed using primary antibodies for GFP (sc-9996, dilution ratio 1:200, Santa Cruz Biotechnology), anti-collagen type II (ab34712, dilution ratio 1:200, Abcam), and type I collagen (NBP2-46874, dilution ratio 1:100, Novus Biologicals, CO, USA). Secondary antibodies were Goat Anti-Mouse IgG H&L DyLight 488 (ab96879, dilution ratio 1:500, Abcam) and Goat anti-Rabbit lgG (H + L) DyLight 594 (35561, dilution ratio 1:1000, Invitrogen). Quantitative histomorphological analysis was carried out by five blinded researchers to evaluate osteochondral regeneration using the modified O’Driscoll scoring system^57^.

### Micro-CT evaluation for osteochondral defect healing

For morphological observation, the specimens were investigated using desktop micro-CT (Bruker, MA, Belgium). A 360-degree scan was carried out at a voltage of 90 kV, a current of 160 μA, and an exposure time of 2 min. Scanned images were aligned using a data viewer (Bruker, MA, Belgium, Ver. 1.5.4.0). From the micro-CT data set, a cylindrical region of interest (ROI) (diameter 2 mm, height 1.5 mm) corresponding to the original defect location was selected for analysis. The bone volume fraction was estimated by bone volume per tissue volume (BV/TV, %) using MeshLab (ISTI-CNR research center, Pisa, Italy, Ver. 2020.03). All images were subsequently reconstructed with the Blender software (Blender foundation, Amsterdam, Netherlands) to generate three-dimensional structures.

### Statistical analysis

Statistical analyses were performed using SPSS version 21 (IBM Corporation, NY, USA). All quantitative data were shown as the mean ± standard derivation (SD). Differences between groups were evaluated by Student’s *t*-test. The results were considered statistically significant at a *p* value of 0.05.

### Reporting summary

Further information on research design is available in the Nature Research Reporting Summary linked to this article.

## Supplementary information

Reporting Summary

Supplementary Information

## Data Availability

All data supporting the conclusions of this study are either provided in this published paper (and its Supplementary Information files) or available from the authors upon reasonable request.
