# Supplementary material for: In-situ forming injectable GFOGER-conjugated BMSCs-laden hydrogels for osteochondral regeneration
Source: NPJ Regen Med. 2023 Jan 6;8:2. doi: 10.1038/s41536-022-00274-z (PMC9822921; doi:10.1038/s41536-022-00274-z)
Supplement: Supplementary file 1 — Reporting Summary [file 41536_2022_274_MOESM1_ESM.pdf]

nature portfolio

Corresponding author(s): Heung Jae Chun

Last updated by author(s): Nov 24, 2022

Reporting Summary

Nature Portfolio wishes to improve the reproducibility of the work that we publish. This form provides structure for consistency and transparency in reporting. For further information on Nature Portfolio policies, see our Editorial Policies and the Editorial Policy Checklist.

Statistics

For all statistical analyses, confirm that the following items are present in the figure legend, table legend, main text, or Methods section.

n/a	Confirmed	
☐	☒	The exact sample size (*n*) for each experimental group/condition, given as a discrete number and unit of measurement
☐	☒	A statement on whether measurements were taken from distinct samples or whether the same sample was measured repeatedly
☐	☒	The statistical test(s) used AND whether they are one- or two-sided<br>*Only common tests should be described solely by name; describe more complex techniques in the Methods section.*
☒	☐	A description of all covariates tested
☒	☐	A description of any assumptions or corrections, such as tests of normality and adjustment for multiple comparisons
☐	☒	A full description of the statistical parameters including central tendency (e.g. means) or other basic estimates (e.g. regression coefficient) AND variation (e.g. standard deviation) or associated estimates of uncertainty (e.g. confidence intervals)
☒	☐	For null hypothesis testing, the test statistic (e.g. *F*, *t*, *r*) with confidence intervals, effect sizes, degrees of freedom and *P* value noted<br>*Give P values as exact values whenever suitable.*
☒	☐	For Bayesian analysis, information on the choice of priors and Markov chain Monte Carlo settings
☒	☐	For hierarchical and complex designs, identification of the appropriate level for tests and full reporting of outcomes
☒	☐	Estimates of effect sizes (e.g. Cohen's *d*, Pearson's *r*), indicating how they were calculated

Our web collection on statistics for biologists contains articles on many of the points above.

Software and code

Policy information about availability of computer code

Data collection	LSM 510 Meta imaging system (Carl Zeiss) and ZEN Black (Carl Zeiss) software were used for image acquisition.
Data analysis	Images were captured and analyzed using the Luminescent Image Analysis System (LAS-4000), Image Studio Lite software (LI-COR Biosciences) and Image J software (NIH).<br>MICRO-CT images were reconstructed Blender software (Blender foundation).

For manuscripts utilizing custom algorithms or software that are central to the research but not yet described in published literature, software must be made available to editors and reviewers. We strongly encourage code deposition in a community repository (e.g. GitHub). See the Nature Portfolio guidelines for submitting code & software for further information.

Data

Policy information about availability of data

All manuscripts must include a data availability statement. This statement should provide the following information, where applicable:

- Accession codes, unique identifiers, or web links for publicly available datasets
- A description of any restrictions on data availability
- For clinical datasets or third party data, please ensure that the statement adheres to our policy

All data supporting the conclusions of this study are either provided in this published paper (and its Supplementary Information files) or available from the authors upon reasonable request.

Human research participants

Policy information about studies involving human research participants and Sex and Gender in Research.

Reporting on sex and gender	n/a
Population characteristics	n/a
Recruitment	n/a
Ethics oversight	n/a

Note that full information on the approval of the study protocol must also be provided in the manuscript.

Field-specific reporting

Please select the one below that is the best fit for your research. If you are not sure, read the appropriate sections before making your selection.

☒ Life sciences ☐ Behavioural & social sciences ☐ Ecological, evolutionary & environmental sciences

For a reference copy of the document with all sections, see nature.com/documents/nr-reporting-summary-flat.pdf

Life sciences study design

All studies must disclose on these points even when the disclosure is negative.

Sample size	Sprague-Dawley (SD) male rats (9-10 weeks old) were randomly divided in to five groups: the control group (n = 2), the defect group (n = 5), the MPEG-PCL group (n = 5), the GFOGER-conjugated group (GFOGER0.8-PEG-PCL; n = 5), and the GFOGER-conjugated PEG-PCL with BMSCs group (GFOGER0.8-PEG-PCL+BMSCs; n = 5).
Data exclusions	No data were excluded from the analysis.
Replication	All attempts at replication were successful.
Randomization	Sprague-Dawley (SD) male rats (9-10 weeks old) were randomly divided in to five groups: the control group (n = 2), the defect group (n = 5), the MPEG-PCL group (n = 5), the GFOGER-conjugated group (GFOGER0.8-PEG-PCL; n = 5), and the GFOGER-conjugated PEG-PCL with BMSCs group (GFOGER0.8-PEG-PCL+BMSCs; n = 5).
Blinding	Histological assessment, quantification was performed by an experimenter blinded to treatment.

Reporting for specific materials, systems and methods

We require information from authors about some types of materials, experimental systems and methods used in many studies. Here, indicate whether each material, system or method listed is relevant to your study. If you are not sure if a list item applies to your research, read the appropriate section before selecting a response.

Materials & experimental systems

n/a	Involved in the study	
☐	☒	Antibodies
☒	☐	Eukaryotic cell lines
☒	☐	Palaeontology and archaeology
☐	☒	Animals and other organisms
☒	☐	Clinical data
☒	☐	Dual use research of concern

Methods

n/a	Involved in the study	
☒	☐	ChIP-seq
☒	☐	Flow cytometry
☒	☐	MRI-based neuroimaging

Antibodies

Antibodies used	FAK (1:200; Santa Cruz Biotechnology; sc-558)<br>pFAK (1:200; Santa Cruz Biotechnology; sc-16662)<br>ERK (1:250; Abcam; ab9363)<br>pERK (1:1000; Bio Legend; 919301)

p38 (1:1000; Abcam; ab31828)
pp38 (1:1000; Abcam; ab4822)
GAPDH (1:1000; Thermo Fisher Scientific; MA5-15738)
Anti-mouse IgG-HRP (1:1000; Cell signaling; 7076)
Goat anti-rabbit IgG-HRP (1:5000; Santa Cruz Biotechnology; sc-2004)
Goat anti-mouse IgG-HRP(1:5000; Santa Cruz Biotechnology; sc-2005)
Rabbit anti-goat lgG H&L-HRP(1:5000; Abcam; ab6741)
Anti-Integrin beta 1 (1:100; Abcam; ab78502)
Integrin α2 (1:100; Santa Cruz Biotechnology; sc-53353)
Integrin α11 (1:100; Santa Cruz Biotechnology; sc-390091)
Goat anti-mouse IgG H&L-DyLight 488 (1:500; Abcam; ab96879)
goat anti-mouse IgG H&L (1:500; Abcam; ab97147)
GFP (1:200; Santa Cruz Biotechnology; sc-9996)
Anti-collagen type II (1:200; Abcam; ab34712),
Collagen I (1:100; Novus Biologicals; NBP2-46874)
Goat anti-Rabbit lgG (H+L) DyLight 594 (1:1000; Invitrogen; 35561)

Validation

Data provided in the manuscript.

Animals and other research organisms

Policy information about studies involving animals; ARRIVE guidelines recommended for reporting animal research, and Sex and Gender in Research

Laboratory animals	Sprague-Dawley (SD) male rats (9-10 weeks old) were used in experiment.
Wild animals	n/a
Reporting on sex	n/a
Field-collected samples	n/a
Ethics oversight	The animal experimental procedures were approved by the Institutional Animal Care and Use Committee (IACUC) in Yeouido St Mary's Hospital of the Catholic University of Korea (YEO-2019011-FA). All animal procedures were performed in accordance with the Animal Protection Act, the Guide for the Care and Use of Laboratory Animals for rodent experiment of provided by the IACUC in Yeouido St Mary's Hospital of the Catholic University of Korea.

Note that full information on the approval of the study protocol must also be provided in the manuscript.
