# Supplementary material for: In-situ forming injectable GFOGER-conjugated BMSCs-laden hydrogels for osteochondral regeneration
Source: NPJ Regen Med. 2023 Jan 6;8:2. doi: 10.1038/s41536-022-00274-z (PMC9822921; doi:10.1038/s41536-022-00274-z)

Supplementary information

In-situ forming injectable GFOGER-conjugated BMSCs-laden hydrogels for osteochondral regeneration

Mi Yeon Ha[1,2,4], Dae Hyeok Yang[2,4], Su Jung You[2], Hyun Joo Kim[2] and Heung Jae Chun[1,2,3,*]

[1]*Department of Biomedicine & Health Sciences, College of Medicine, The Catholic University of Korea, Seoul 06591, Republic of Korea*

[2]*Institute of Cell and Tissue Engineering, College of Medicine, The Catholic University of Korea, Seoul 06591, Republic of Korea*

[3]*Department of Medical Life Sciences, College of Medicine, The Catholic University of Korea, Seoul 06591, Republic of Korea*

[4]*These authors contributed equally: Mi Yeon Ha, Dae Hyeok Yang.*

Email: aldus6364@catholic.ac.kr; yangdh@catholic.ac.kr

*** Corresponding author. Heung Jae Chun**

Institute of Cell and Tissue Engineering, College of Medicine, The Catholic University of Korea, 222, Banpo-daero, Seocho-gu, Seoul, 06591, Republic of Korea

Tel: +82 2 2258 7033, Fax: +82 2 2258 7494, Email: chunhj@catholic.ac.kr

Supplementary Figure 1: Surgical procedures for osteochondral defect repair in the rat model step by step.

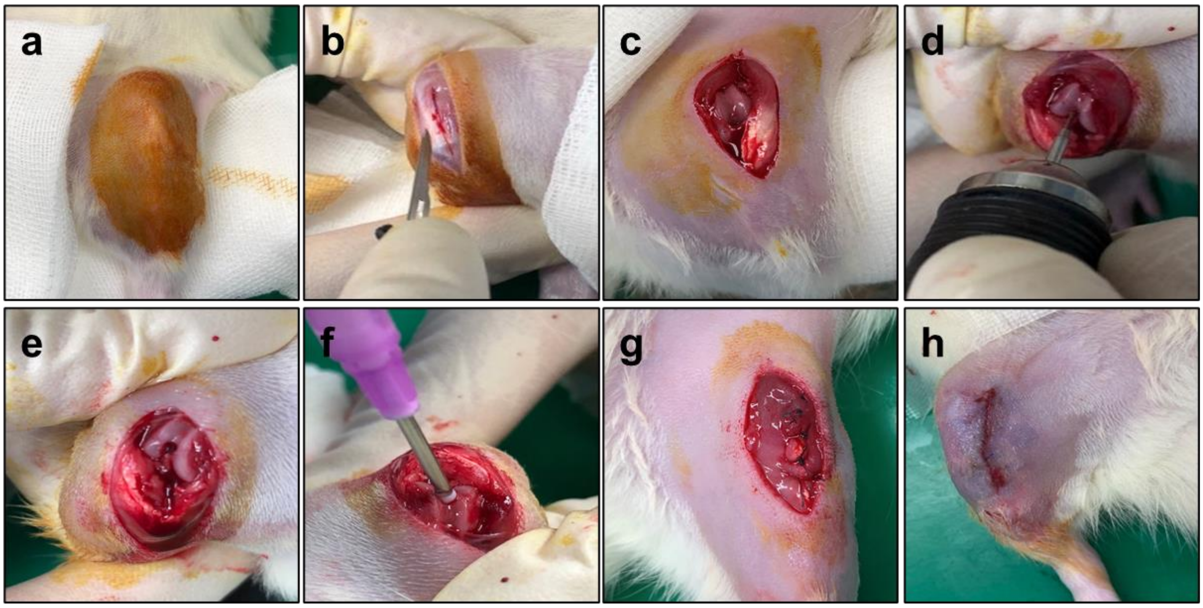

a After shaving of the rat; **b** Cutting the skin of the rat; **c** The patella was exposed; **d** and **e** Defect site was 2 mm in diameter by drilling; **f** Hydrogel was implanted into the defect site; **g** and **h** Skin was closed with a 5-0 absorbable suture and tissue adhesive bond.

Supplementary Figure 2: Evaluation of integrin expression and cell morphology induced by hydrogel formulations at day 3.

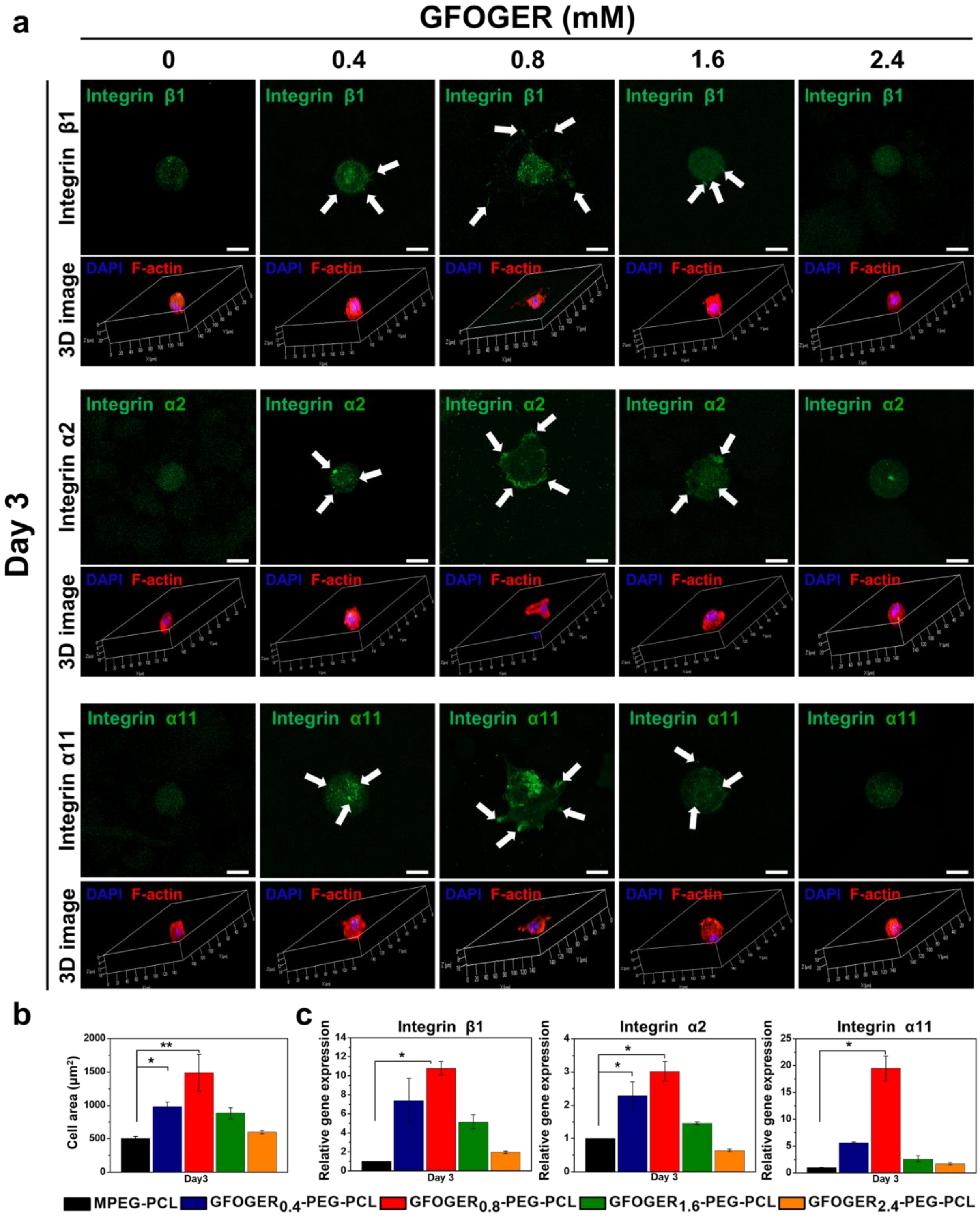

a Immunofluorescence assay of integrins β1, α2 and α11 expressions (green, white arrows), F-actin (red) and nucleus (blue) (scale bar, 5 μm). And confocal laser scanning microscope 3D images of a cell morphology in hydrogels. **b** Quantification of cell spreading area analyzed based on 3D cell images of a cell morphology in hydrogels. **c** mRNA expressions of integrin β1, α2 and α11 of BMSCs cultured in hydrogels for 3 days (Data in (b and c) are shown as mean ± SD, unpaired Student's *t*-test. $^{*}p < 0.05$ and $^{**}p < 0.01$).

Supplementary Figure 3: Evaluation of integrin expression and cell morphology induced by hydrogel formulations at day 7.

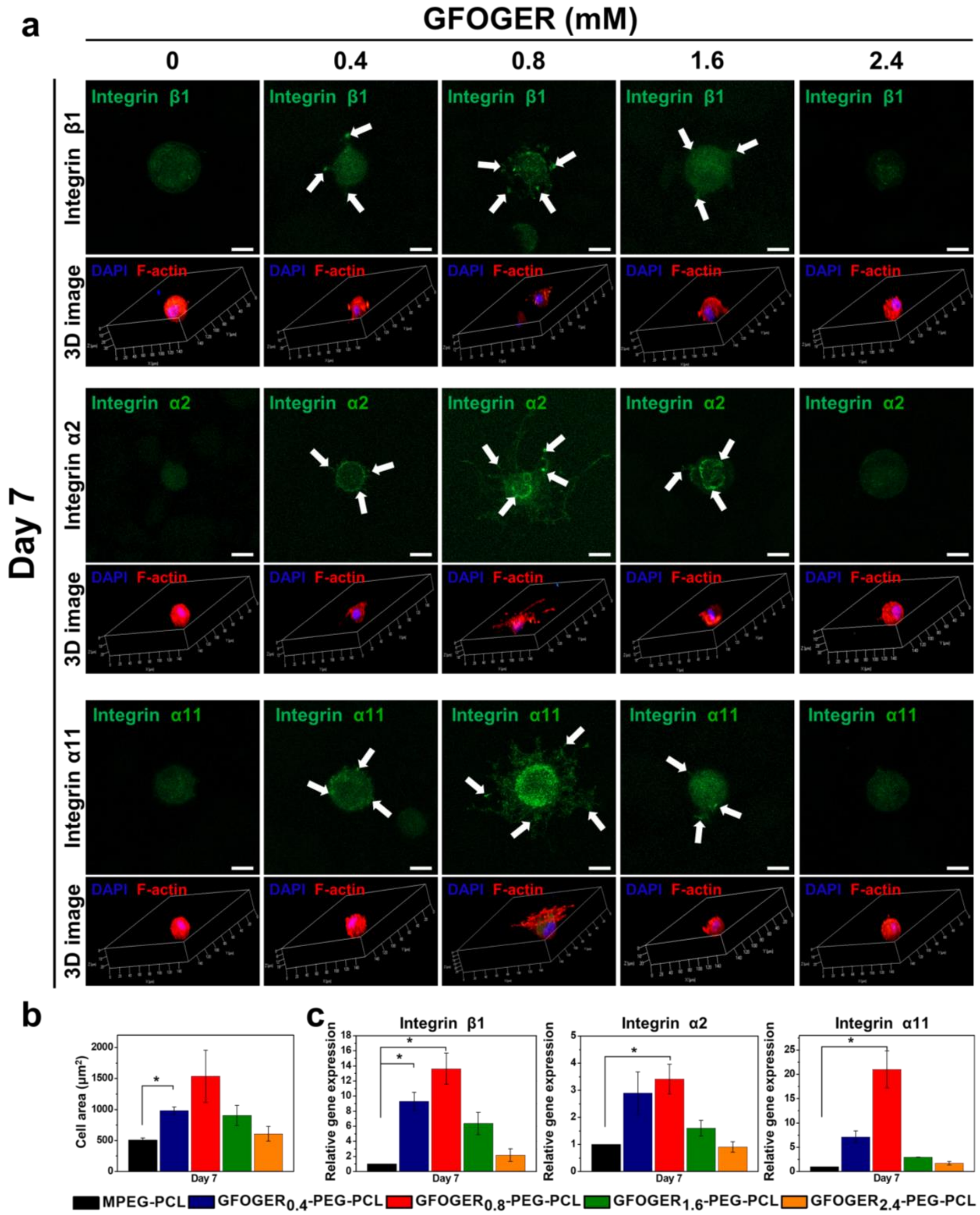

a Immunofluorescence assay of integrins β1, α2 and α11 expressions (green, white arrows), F-actin (red) and nucleus (blue) (scale bar, 5 μm). And confocal laser scanning microscope 3D images of a cell morphology in hydrogels. **b** Quantification of cell spreading area analyzed based on 3D cell images of a cell morphology in hydrogels. **c** mRNA expressions of integrin β1, α2 and α11 of BMSCs cultured in hydrogels for 7 days (Data in (b and c) are shown as mean ± SD, unpaired Student's *t*-test. *$p < 0.05$).

Supplementary Figure 4: Histological analysis of osteochondral repair in rats.

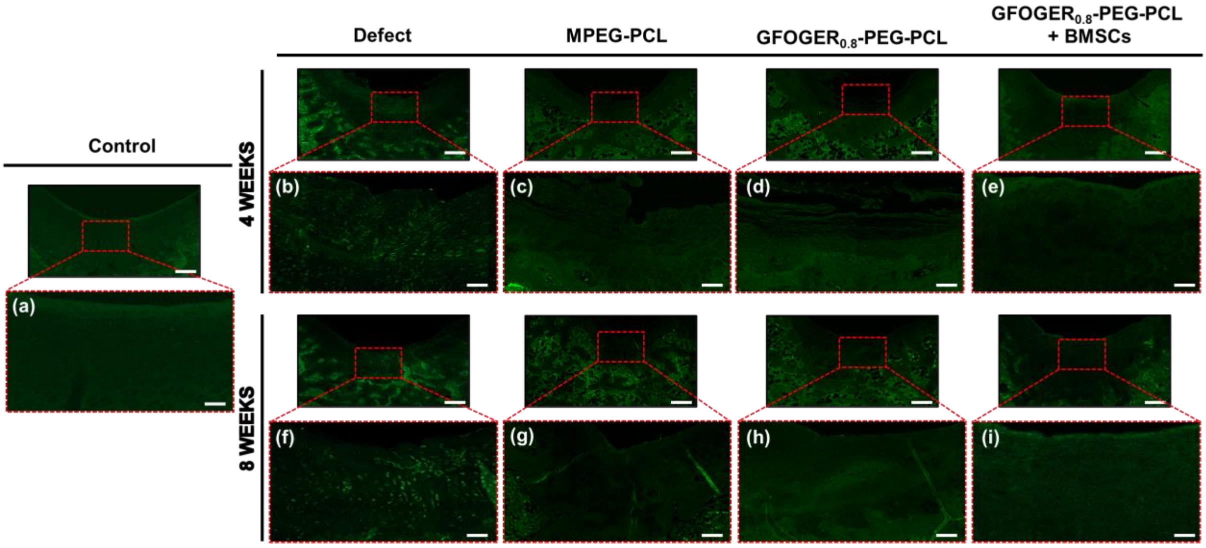

Immunofluorescence type I collagen staining of rat cartilage defect sites after 4 and 8 weeks of repair. Scale bar: 100 and 50 μm. And the red frames indicate the magnified cartilage area ((a)-(i)).

Supplementary Figure 5: Uncropped and unprocessed scans of blots shown in Fig. 5a.

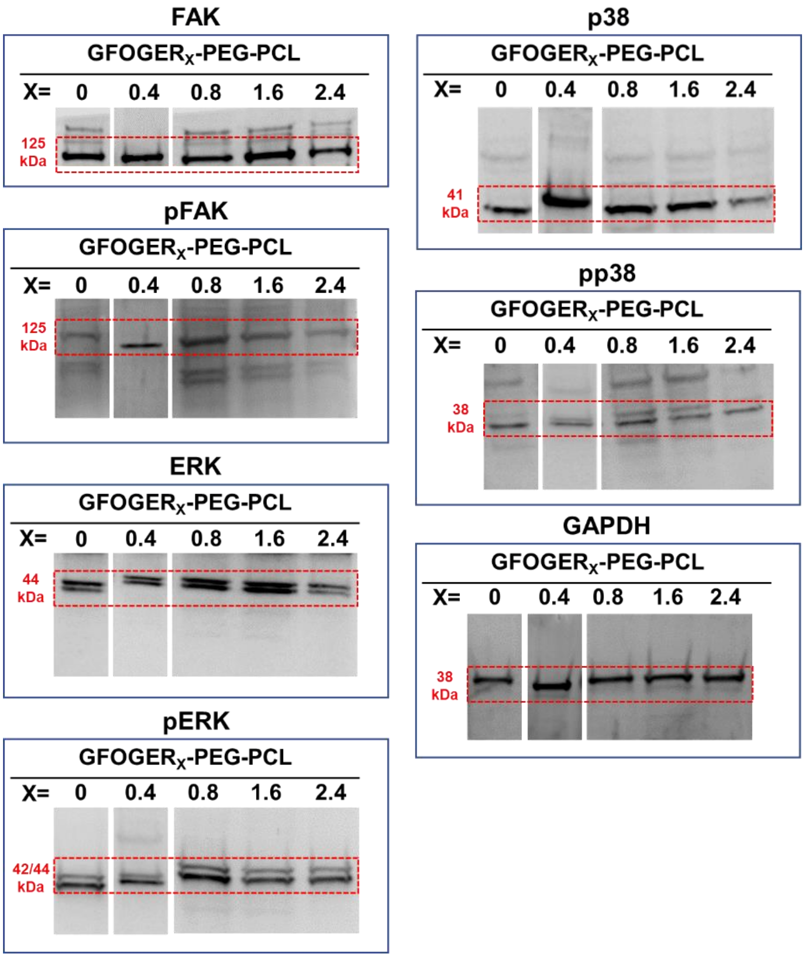

Supplement: Supplementary file 2 — Supplementary Information [file 41536_2022_274_MOESM2_ESM.pdf]
